# sGC redox regulation and asthma

**DOI:** 10.1186/2050-6511-16-S1-A26

**Published:** 2015-09-02

**Authors:** Dennis J Stuehr, Arnab Ghosh, Cynthia J Koziol-White, Kewal Asosingh, Georgina Cheng, Lisa Ruple, Jennifer Rodgers, Dieter Groneberg, Andreas Friebe, Johannes-Peter Stasch, Reynold A Panettieri, Mark A Aronica1, Serpil C Erzurum

**Affiliations:** 1 Department of Pathobiology, Lerner Research Institute, Cleveland Clinic, Cleveland, Ohio 44195, USA; 2 University of Pennsylvania Medical Center, Pulmonary, Allergy, and Critical Care Division, Philadelphia, PA 19104, USA; 3 Institute of Vegetative Physiology, Universität Würzburg, D-97070 Würzburg, Germany; 4 Bayer Pharma AG, Aprather Weg 18a, D-42096 Wuppertal, Germany; 5 Respiratory Institute, Cleveland Clinic, Cleveland, Ohio 44195, USA

## 

Asthma is defined by airway inflammation and hyper-responsiveness, and contributes to morbidity and mortality worldwide. Although bronchodilation is a cornerstone of asthma treatment, current bronchodilators become ineffective with time and with worsening asthma severity. We investigated an alternative pathway for bronchodilation that involves activating the airway smooth muscle enzyme, soluble guanylate cyclase (sGC).

The pharmacologic sGC stimulator BAY 41-2272 or a NO donor each triggered a dose-dependent bronchodilation in precision cut human lung slices from healthy donors and in mouse tracheal rings, whereas BAY 60-2770 was a less effective bronchodilator in these circumstances. Neither NO nor BAY 41-2272 relaxed tracheal rings obtained from sGC-/- mice. This established the NO-sGC pathway can trigger bronchodilation in healthy airway. In live animal studies, a single intra-tracheal administration of either BAY 41-2272 or BAY 60-2770 reversed the airway hyper-responsiveness that had developed in mice with allergic asthma and restored their normal lung function. The sGC recovered from the mouse asthmatic lungs displayed three hallmarks of oxidative damage that render it NO-insensitive, and identical changes were found to occur in sGC obtained from human lung slices or from cultured cells that had been exposed to low chronic NO. Our findings reveal how allergic inflammation in asthma, and its resulting airway inflammation and excessive NO production, impede the natural NO-sGC-based bronchodilation pathway, and suggest that pharmacologic sGC stimulants or activators can provide a novel therapeutic approach to achieve bronchodilation despite this loss.

